# The declines of heterogeneity and stability in diatom communities are associated with human activity

**DOI:** 10.1002/ece3.10695

**Published:** 2023-11-01

**Authors:** Rong Wang, Wenxiu Zheng, Min Xu, Hui Yang

**Affiliations:** ^1^ State Key Laboratory of Lake Science and Environment, Nanjing Institute of Geography and Limnology Chinese Academy of Sciences Nanjing China; ^2^ The Fuxianhu Station of Plateau Deep Lake Research, CAS Yuxi China; ^3^ The Fuxianhu Station of Plateau Deep Lake Field Scientific Observation and Research Yuxi China; ^4^ College of Urban and Environmental Sciences Hubei Normal University Huangshi China; ^5^ State Key Laboratory of Palaeobiology and Stratigraphy, Nanjing Institute of Geology & Palaeontology Chinese Academy of Sciences Nanjing China; ^6^ School of Mathematics and Physics Anhui University of Technology Ma'anshan China

**Keywords:** biodiversity, ecological network, lake, network distance, richness, species turnover

## Abstract

Anthropogenic forcing caused the biodiversity loss and stability decline of communities. There is still controversy over whether the decline in biodiversity will lead to a decrease in community stability. The stability of biological communities is related to both biodiversity and structure, and this paper aims to reveal the human impacts on diatom communities' biodiversity and structure. We studied the richness, *β*‐diversity and network distance of diatom communities in Qinghai‐Xizang, Yunnan‐Sichuan and Lower Yangtze River Basin, China through empirical dataset and simulation method. The results showed that the diatoms richness in the Qinghai‐Xizang and the Yunnan‐Sichuan region was lower and the network distance was higher than that of the Lower Yangtze River Basin. *β*‐diversity in the Lower Yangtze River Basin was the lowest and the diatom network distance responds negatively to human population densities in China. The simulation showed that the network distance kept constant during random species loss, and declined while specialist species were lost or replaced by generalist species. The results suggested diatom communities' homogeneity and stability decline were associated with human activities. Human impacts may cause biodiversity loss targeted to specialist species or no biodiversity loss while generalist species replace those specialist species. This study showed that how diversity changes determined ecological stability depends on the type of species changes.

## Graphical abstract

This paper revealed how biodiversity change will cause resilience decline based on a large diatom dataset (273 lakes and 452 species) in China. It introduced a network science method to evaluate the diatom structure across environmental gradients, and simulated changes in biodiversity—including species loss and turnover—using the diatom dataset as a reference condition to observe the response of diatom's structure along changes in biodiversity. This study showed that how diversity changes determined ecological resilience depends on the type of species changes.

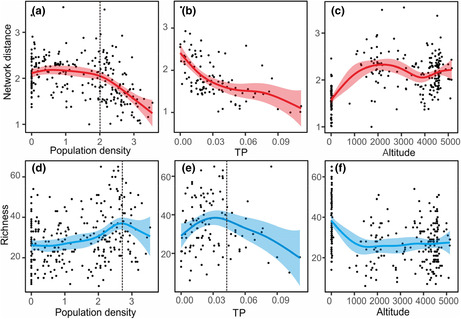

## 1 INTROUDUCTION

Studies have found considerable declines in biodiversity (IPBES, 2019) and loss of ecological stability at both global and regional scales, which reflect land and water conversion to agricultural and industrial uses and invasions of exotic species, among other drivers (Steffen et al., 2011). Assessing the impact of human activities on biology communities is beneficial for us to better protect biodiversity and maintain ecosystem stability. In addition, despite an abundance of studies on various aspects and scales, there is a lack of consensus about the relationships between biodiversity and ecological stability (Hooper et al., 2005; Ives & Carpenter, 2007; Pennekamp et al., 2018).

Long‐lasting anthropogenic forcing will cause species invasion, species loss or species turnover in biological communities (Buckley & Jetz, 2008), and these changes in biodiversity can impact stability in response to changes in external perturbations (Chapin III et al., 2000; Oliver et al., 2015). Ecological stability consists of numerous components, including temporal variability, resistance to environmental change and rate of recovery from disturbance (Pennekamp et al., 2018). In the ecosystem level, studies suggest that biodiversity loss could disturb ecological stability (Cardinale et al., 2012; Ives & Carpenter, 2007), but others conclude that the impact of loss of biodiversity on stability depends on the type of species that is lost (Chen et al., 2000; Sasaki & Lauenroth, 2011). For example, the removal of the keystone species is more likely to trigger the collapse of an ecosystem (Dunne, 2006; Proulx et al., 2005). In addition, species turnover means one species can replace another under environmental pressure so that the biodiversity will keep constant, but the community's stability may be affected if species with different functions take over (Doncaster et al., 2016). These factors indicate that in order to understand how changes in biodiversity affect ecological stability, it is necessary to understand what species are responsible for which changes in biodiversity (Hooper et al., 2005).

Most studies use a single component of a system, such as biomass (Pennekamp et al., 2018), to evaluate system's level of ecological stability. The structure of a community is considered to be an important factor in its stability (Scheffer et al., 2012), but this can be difficult to measure. Network science simplifies the interactions of components such as biological species, computers and airports within different systems (Griffith et al., 2018; Kay et al., 2018) by providing parameters to measure the structure of the system. Albert et al. (2000) tested the resilience of various networks to ‘failure’ and ‘attack’ perturbations in social systems, which may respectively correspond to removing species in either a random or targeted manner in biological communities. They found that a strongly hierarchical system such as a scale‐free network which is one with a power‐law degree distribution displays a surprisingly high degree of tolerance against random failures, but was more vulnerable to a targeted attack. The ecosystem is always a hierarchical structure system, in which every ecological component interacts with each other. Thus, it implied that the biological community structure would respond differently to various changes in biodiversity and that the parameters of network structure may help us understand how biodiversity change affects the biological community's stability in greater depth.

Network parameters such as network skewness (Wang, Dearing, et al., 2019), network heterogeneity (Xu et al., 2022) and network distance (Barberán et al., 2012; Yuan et al., 2021) have already been employed to identify the structure of ecological communities. These parameters are mainly calculated based on the species degree, that is, the number of linked other species in the communities, or species connectivity, that is, the links among species. In Wang, Dearing, et al., 2019, it mainly focussed on network skewness, which is a metric that describes the distribution of species degree in a diatom network. Other studies, such as Albert et al. (2000), Barberán et al. (2012) and Yuan et al. (2021), focused on network distance, which describes the average length of all the shortest paths (geodesics) between all pairs of connected species. This means that communities with only large‐degree species will have a shorter average distance than communities with only small‐degree species. A short‐distance community describes a community in which the species are functionally similar, while a long‐distance community is a community in which the species are functionally dissimilar. Similar perturbations may impact more species in a short‐distance community than in a long‐distance community, so a short network distance will show low stability to environmental changes. Because network distance is more used to evaluate the complexity and stability of structures (Albert et al., 2000; Barberán et al., 2012; Yuan et al., 2021), we will focus on network distance in this paper.

Lake ecosystems are seriously affected by humans (Ho et al., 2019; Jane et al., 2021). Many lake ecosystems are currently undergoing critical transitions from a clear to a turbid state, because of human impacts (Scheffer & Jeppesen, 2007). In that sense, understanding how a lake ecosystem losses its stability under anthropogenic forcing is key to the protection of the ecosystem services. Meanwhile, the theory of critical transition is widely tested by using cases from lake ecosystems (Scheffer et al., 2012; Wang et al., 2012), and many of these studies are focused on algae because it is the dominant biological community in turbidity states and algal stability is the key to the state of the whole lake. This paper focuses on diatoms, which constitute one of the main algal communities in lakes. These communities are sensitive to environmental changes and are therefore widely used to monitor and assess water quality in lakes. We studied the distribution of diatoms from West to East China, a geographical location that offers a wide range of gradients relating to environmental and human activities. We checked diversity distributions and used the parameter of network distance to measure the community structure of diatoms in different environments. Finally, we simulated changes in biodiversity—including species loss and turnover—using the diatom dataset as a reference condition to observe the response of network distance along changes in biodiversity. The main aim of this study was to reveal the human impacts on diatom communities and discuss the impacts of biodiversity loss on ecological stability.

## 2 MATERIALS AND METHODS

### 2.1 Study region and samples

**FIGURE 1:**
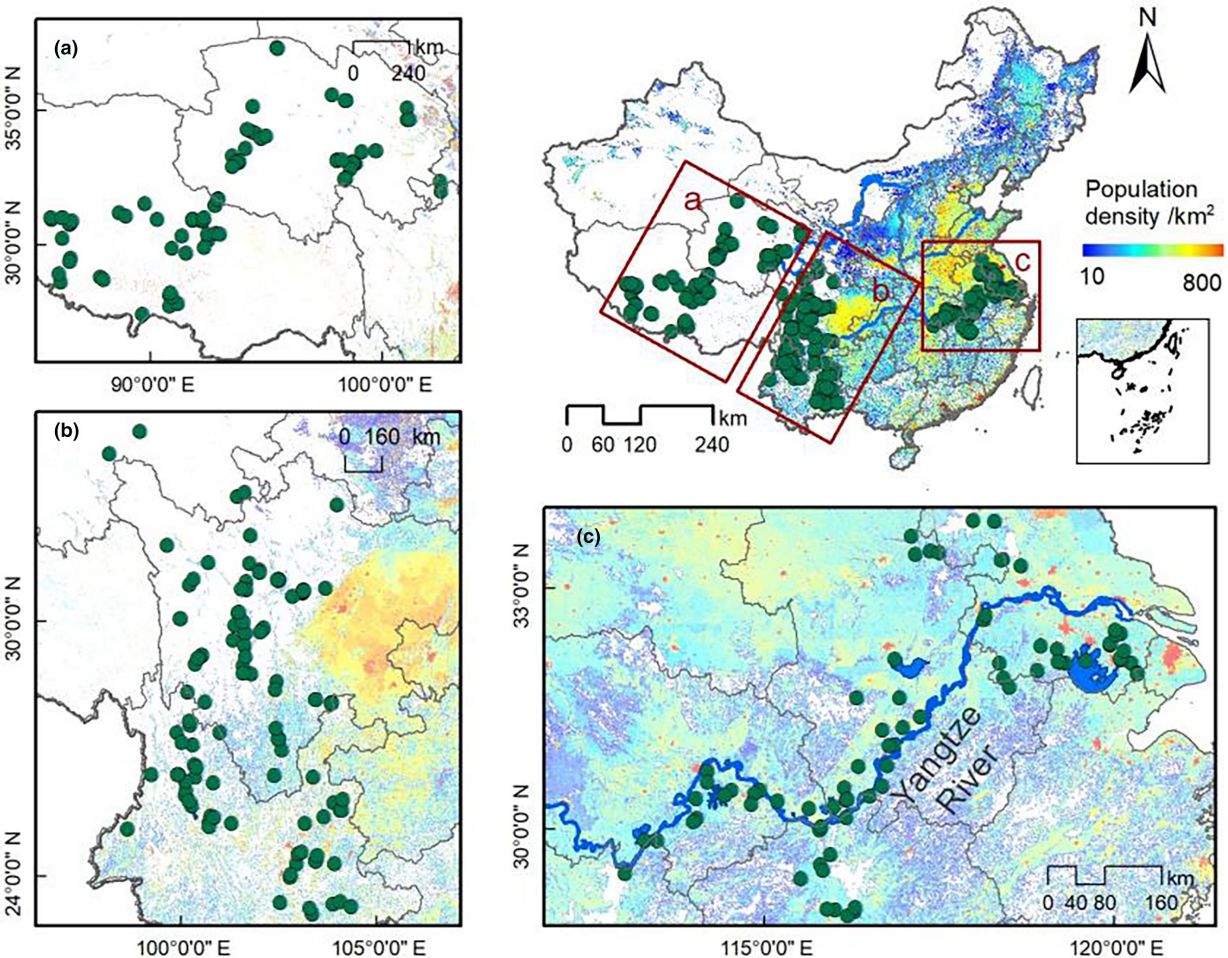
Research sites. (a) Qinghai‐Xizang; (b) Yunnan‐Sichuan; (c) middle and lower Yangtze River basin. The green points are lakes.

The study region takes in three subregions of China. The western region mainly covers the Tibetan plateau including Qinghai and Xizang provinces (Figure 1). This area is mainly set above 4000 m a.s.l. The land cover is mainly pasture, and it has the lowest population density in our study regions. The second region is the areas surrounding Hengduan Mountain, including Yunnan and Sichuan provinces. This region contains varied types of land cover, from mountain to plain and from forest and grassland to agriculture. The lakes' catchments include towns as well as agriculture at low‐altitude regions which have been greatly affected by human activities, and mountain regions which are far from any direct human impact. The third region covers eastern China, mainly the middle and lower Yangtze River basin, which has a long history of human impact going back almost 5000 years. The lakes here are mainly shallow and are significantly polluted by agriculture, fishery, industry and sewage. The water quality in the lakes has declined since the 1970s and the main environmental problem is eutrophication.

We collected water and sediment samples from 273 lakes in the three study regions, and each lake contained one diatom community in this analysis. Water and lake sediment samples were mainly collected from the deepest part of each lake. Water samples were collected from the surface of each lake for chemical analysis. The environmental variables included Secchi depth (SD), pH and water depth were recorded in the field. The environmental variables of water samples including Ca^2+^, SO_4_^2+^, K^+^, Na^+^, Cl^−^ and Mg^2+^ concentrations were analysed for all the samples, and total nitrogen (TN), total phosphorus (TP), were also analysed in Yunnan‐Sichuan and the middle and lower Yangtze basin. Short sediment cores were collected from the deepest parts of each lake using a Kajak gravity corer (Renberg, 1991). The uppermost 1 cm of each sediment core was taken as representative of the contemporary diatom assemblages and kept refrigerated at 4°C prior to analysis. Diatom sample preparations followed standard procedures (Battarbee et al., 2002). For surface samples, around 500 diatom valves were counted from each lake except for a few lakes with sparse diatom valves where the count was reduced to ~300 valves. Nomenclature and taxonomy mainly followed Krammer and Lange‐Bertalot (1988a, 1988b, 1991a, 1991b, 2000). Only diatoms identifiable to species level were counted. More details about sampling, fieldwork and laboratory analysis could be found in Yang et al. (2008), Wang et al. (2011), Yao (2011), Wang, Dearing, et al. (2019) and Wang et al. (2020).

### 2.2 Ordination and biodiversity of diatom communities

The ordination analysis was employed to explore the determined environmental factors for the diatoms' distribution, and we used relative abundance diatom data in the analysis. The gradient length measures the beta diversity in community composition along the ordination axes, and the compositional diatom species in all lakes of this study had a gradient length of 7.3 SD units. The value is >4.0, so we used an unimodal method, that is, the Canonical Correlation Analysis (CCA) for constrained analysis to reveal the relationship between diatom communities and environmental variables. We included all environmental variables except TN and TP because they were absent in this Tibetan plateau. The scales of these variables are different, and thus in the analysis, the relative diatom abundance data were square‐root transformed, and the environmental data were log_10_‐transformed. In order to limit the effect of rare species upon the ordination results, we chose down‐weighted rare species in the analysis. The CCA analysis was calculated in Canoco 5. We calculated diatom richness in each sample as well as the *β*‐diversity for all pairwise combinations of samples in the three regions to assess the dissimilarity of species in each region. Sørensen dissimilarity index is used as an indicator of *β*‐diversity. The Sørensen index was calculated using the ‘vegan’ package in R (Oksanen, 2022).

### 2.3 Network construction and network average distance

Biodiversity metric gives information about how many species are in a sample such as richness or how similarities of different communities such as Sørensen dissimilarity index. Here in order to reveal how these species are linked in a community, we constructed a network using the presence or absence of individual species in the samples. The main task of network construction is to decide whether there is a link between pairs of diatom species. The strength of inter‐specific associations was calculated using 2 × 2 contingency tables for all possible pairings in the dataset, and the association coefficient $V_{\mathrm{ij}}$ for each pair of species *i* and *j* was used to determine the strength of the linkage between pairs as quantified by Cramér's *V*:

(1) $$V_{\mathrm{ij}}=\frac{\mathrm{ad}-\mathrm{bc}}{\sqrt{\left(a+b\right)\left(c+d\right)\left(a+c\right)\left(b+d\right)}}$$

where *a, b, c* and *d* are from the 2 × 2 contingency tables, *a* is the number of lakes with both species *i* and species *j*, *b* is the number of lakes with species *i* but not species *j*, *c* is the number of lakes with species *j* but not species *i*, and *d* is the number of lakes with neither species *i* nor species *j*. *V* range values were set between +1 (strong association) and −1 (strong avoidance), and the analysis of network parameters used only sets of most highly associated pairings (‘connected pairings’), given by the upper quartile (Q3) of positive *V* (*V+)*. Other thresholds of the connected pairings will change the values of network parameters but will not change its relative size (Wang, Dearing, et al., 2019). Thus, we will not discuss the effect of the threshold selection in this paper.

The network distance of community, $\bar{d}$, represents the average length of all of the shortest paths between two connected nodes in the network:

(2) $$\bar{d}=\frac{\sum_{u\neq v}\left\{d\left(u,v\right),\forall u,v\in V\right\}}{N∙\left(N-1\right)}$$

where the numerator is the sum of geodesic distances between pairs of connected nodes, $d\left(u,v\right)$, for all *u*, *v* in network *V* and *N* is the number of nodes in the network. A geodesic path is the shortest possible path between two vertices (Newman, 2010). The geodesic distance is the overall length of the geodesic path, which is measured according to the number of links in that geodesic path.

### 2.4 Simulation of biodiversity change in the ecological networks

We ranked each species in each sample according to their degrees and considered the most connected species as large‐degree species and the least connected species as small‐degree species. A large‐degree species interacts with many others, indicating the occupancy of a wide niche of a resource or habitat generalist. Meanwhile, a small‐degree species interact with few others, indicating the occupancy of a narrow niche as a resource or habitat specialist. External forcing will cause species invasion, species loss or species turnover in biological communities (Buckley & Jetz, 2008), which means the biodiversity can decline, keep constant or increase. To simplify the simulation result, we did not simulate all types of species changes. The purpose of this simulation is to simulate the impact of biodiversity loss on the communities' stability, and thus we mainly simulate three types of species loss. We used ‘failure’ and ‘attack’ experiments in social networks (Albert et al., 2000) to represent the outcome of random species loss and ordered species loss, respectively. We simulated network failure by the random deletion of *n* species from each assemblage, and an attack on the diatom network by the deletion of *n* large‐degree or small‐degree species from each community. In addition, we also simulate a type of species turnover, that is, generalist species replace specialist species. To simulate the species turnover, *n* small‐degree species were replaced by *n* large‐degree species that co‐occur within a lake's diatom assemblage in the full dataset (other than the focal lake). We then observed the corresponding changes in network distance for each simulation. Throughout the process, we set the number of changes (i.e. delete or replace) from 1 to 10 to simulate the increasing pressure in both species loss and turnover.

## 3 RESULTS

### 3.1 Environment variables and diatom distribution across the three regions

There were 273 lakes in our dataset, including 116 lakes in the Yunnan‐Sichuan region, 81 lakes in the Qinghai‐Xizang Plateau, and 76 lakes in the middle and lower reaches of the Yangtze River. There was a significant altitude gradient between the lakes. The altitude of the Qinghai‐Xizang plateau lakes was between 5148 and 2798 m a.s.l., and that of the Yunnan‐Sichuan lakes was between 4778 and 1078 m a.s.l. The lakes in the middle and lower reaches of the Yangtze River had the lowest altitude, at between 789 and 8 m a.s.l. There were obvious differences in human population density in the three regions. The intensity of human activities in the Qinghai‐Xizang Plateau was the lowest, given a population density of 0–97 people per square kilometre in the basin. There are between 0 and 450 people per square kilometre in the Yunnan‐Sichuan region, while the population density in the middle and lower reaches of the Yangtze River was the highest, at 6–3489 people per square kilometre. In terms of water depth, there were deep and shallow lakes in Qinghai‐Xizang Plateau and the Yunnan‐Sichuan region, and the range of water depth was 0.1–78 m and 0.25–134 m respectively. The lakes in the middle and lower reaches of the Yangtze River are much shallower, with water depths ranging from 0.2 to 13 m. In terms of chemical indicators, there were both freshwater and salt lakes in the Qinghai‐Xizang Plateau and the variation in water conductivity was 100–119,400 μs/cm. The lakes in Yunnan‐Sichuan and the middle and lower reaches of the Yangtze River were freshwater, and the variation range of water conductivity was 5–700 μs/cm and 60–852 μs/cm respectively.

**FIGURE 2:**
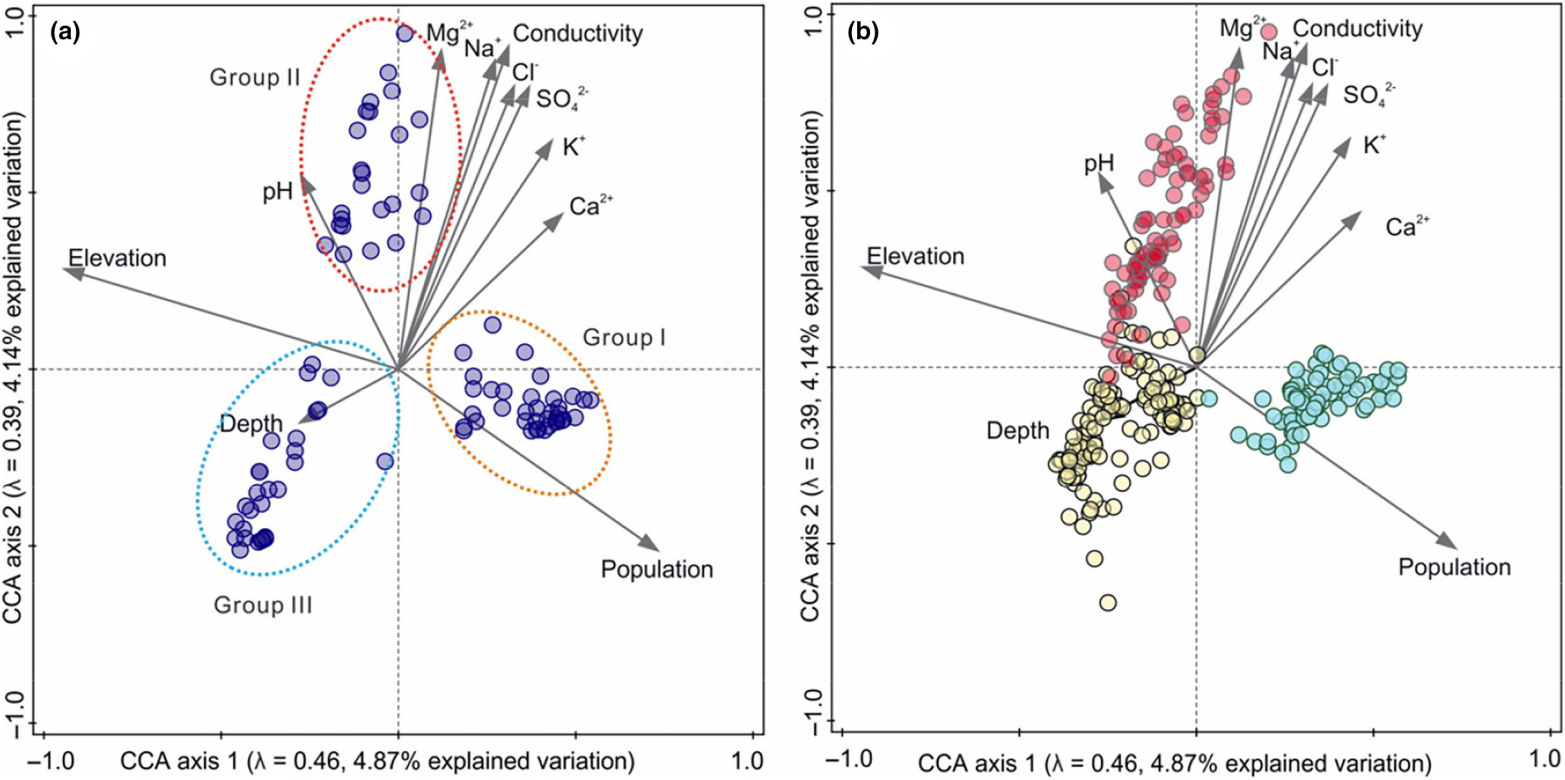
CCA analysis of the effect of environment on diatom species (a) and diatom communities (b). The points in the figure are samples. The 89 species with the largest fit into the ordination space are shown in (a). In (b), the blue points are the 76 lakes in the middle and lower reaches of the Yangtze River, the red points are the 81 lakes of the Qinghai‐Xizang Plateau and the yellow points are the 116 lakes of the Yunnan‐Sichuan region.

A total of 452 diatom species were identified across the three regions, of which *Nitzschia palea* was the most widely distributed diatom species, appearing in 153 lakes. *Fragilaria pinnata*, *Aulacoseira alpigena*, *Achnanthes marginulata*, *Navicula cryptotenella*, *Navicula pupula* var. *pupula*, *Cyclotella ocellata*, *Achnanthes minutissima var. minutissima*, *Cyclotella pseudostelligera* were also widely distributed, appearing in more than 100 lakes. There were 263 diatoms species in the 81 lakes of the Qinghai‐Xizang Plateau, 322 diatoms species in the 116 lakes of the Yunnan‐Sichuan region and 200 diatoms species in the 76 lakes in the middle and lower reaches of the Yangtze River. Figure 2 shows the CCA analysis of the diatom community and other environmental factors. The first two axes explained 4.87% and 4.14% of variation, and the eigenvalues for the first two axes were 0.46 and 0.39. Diatom species could be divided into three categories: species related to high human population density (Group I), species with high conductivity and a shallow water environment (Group II) and species with low conductivity and a deep‐water environment (Group III). Figure 2b shows that the main environmental gradients that determined differences in diatom communities were human population and altitude.

### 3.2 Diatom diversity and average distance in three regions

**FIGURE 3:**
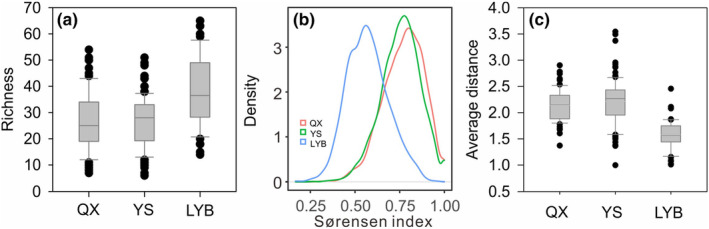
Biodiversity index and network distance across the three regions. (a) richness; (b) density distribution of pairwise Sørensen index in the three regions; (c) average distance of diatom network. In all plots, QX, Qianghai‐Xizang; YS, Yunnan‐Sichuan and LYB, middle and lower Yangtze River basin.

Figure 3 shows the differences in diatom richness and network distance in the dataset. Paired *t*‐test between richness in QX and LYB gave a *t*‐value −5.76 and *p* < .0001, and paired *t*‐test between richness in YS and LYB gave a *t*‐value −6.57 and *p* < .0001. It suggested that the species richness of diatom communities in the Qinghai‐Xizang Plateau and the Yunnan‐Sichuan region was lower than that of the middle and lower reaches of the Yangtze River. However, the *β*‐diversity of the community in the middle and lower reaches of the Yangtze River was the lowest. The Sørensen index of Qinghai‐Xizang Plateau had a mean value of 0.77 with a standard deviation of 0.11, and the Sørensen index of Yunnan‐Sichuan had a mean value of 0.76 with a standard deviation of 0.11, and the Sørensen index of Yunnan‐Sichuan had a mean value of 0.57 with a standard deviation of 0.12. The Sørensen index of Qinghai‐Xiang was similar to that of Yunnan‐Sichuan (Figure 3b) and was larger than in the middle and lower Yangtze River basin. The network distance in QX ranged from 1.4 to 2.9 with a median value at 2.1. The network distance in YS was ranged from 1 to 3.5 with a median value at 2.3, and the network distance in LYB is ranged from 1.0 to 2.5 with a median value at 1.6. Paired *t*‐test between the average distance in QX and LYB gave a *t*‐value 12.23 and *p* < .0001, and paired *t*‐test between the average distance in YS and LYB gave a *t*‐value of 12.05 and *p* < .0001. The network distance was higher in the Qinghai‐Xizang Plateau and Yunnan‐Sichuan than it was in the middle and lower reaches of the Yangtze River. This indicated that the heterogeneity of the diatom community in the middle and lower reaches of the Yangtze River was low.

### 3.3 Changes in diatom richness and network distance along environmental variables

**FIGURE 4:**
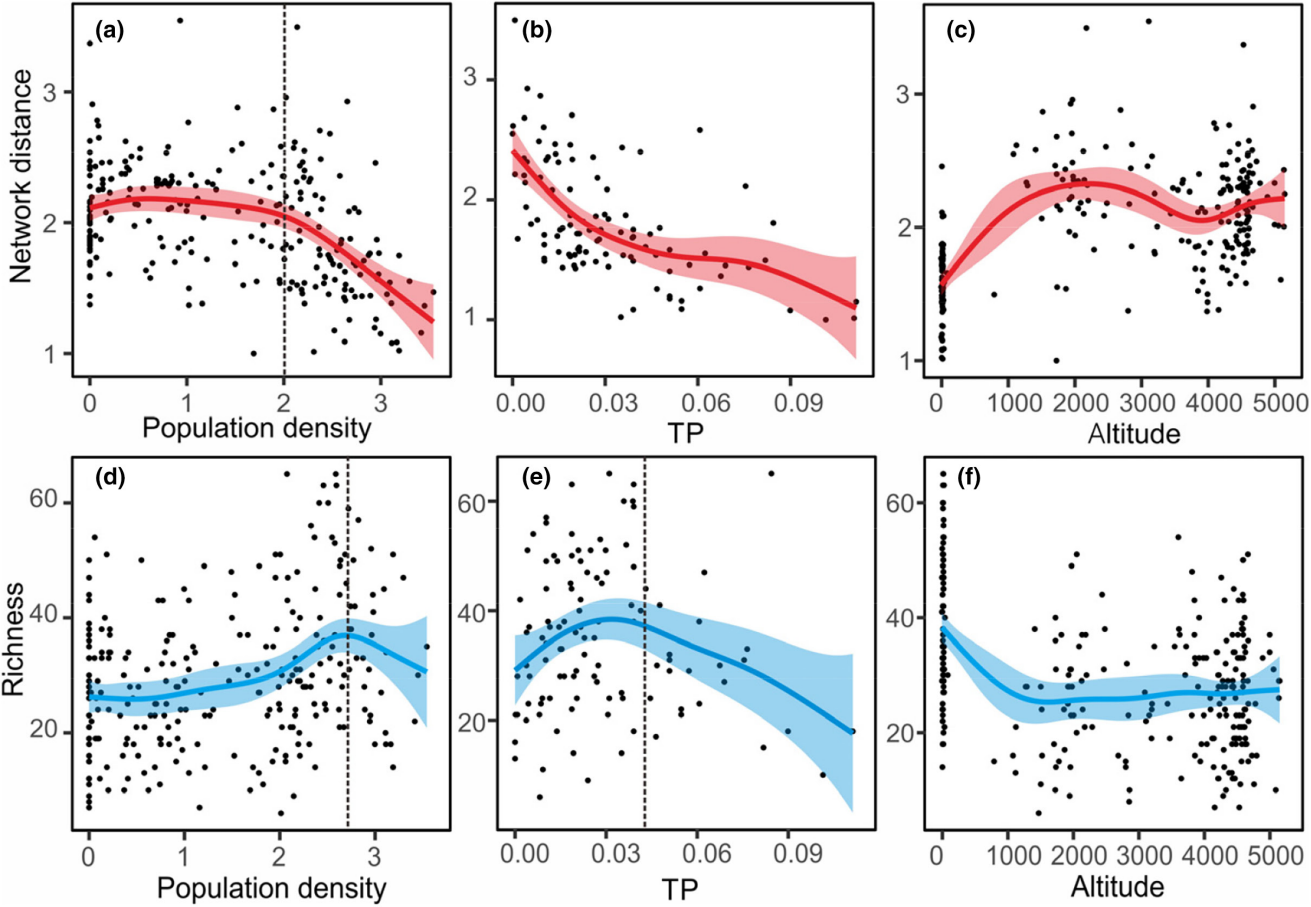
The relationship between species richness, network distance and main environmental factors. It showed the relationships between network distance with population density (a), TP (b), altitude (c) and the relationships between richness with population density (d), TP (e), altitude (f). The population density was log10(*x* + 1) transformed. The scatters were the real data, the smooth curves were the fitting lines by the generalised additive model (GAM) and the shades with the same colour represent the 95% confidence interval. The dashed line in (a) represents a human population density value of around 100 people per km^2^. The dashed line in (d) represented a human population density value of around 500 people per km^2^. The dashed line in (e) represented a TP value at 0.045 mg/L.

We compared diatom richness and average distance with the gradient of human activities, total phosphorus and altitude (Figure 4). We found that both diatom richness and average distance showed a nonlinear correlation with population density in lake's catchment. Below a population density of 500 people per km^2^, increasing population density corresponded to rising diatom richness, but above this value, the diatom richness showed a downward trend with an increase in population density (Figure 4d). When the population density was lower than 100 people per km^2^, the average distance was stable and became lower and lower as population density increased from 100 people per km^2^ (Figure 4a). Figure 4b,e show the relationships between the indicators and total phosphorus (TP). As a significant result of human impacts, TP in the lake showed a good correlation with network distance but a nonlinear correlation with richness. The richness increased in the low value of TP (<0.045 mg/L) but decreased in the high value (>0.045 mg/L). In contrast, we found the impacts of altitude on richness and network distance were not direct (Figure 4c,f).

### 3.4 Diatom network change under simulated biodiversity change

**FIGURE 5:**
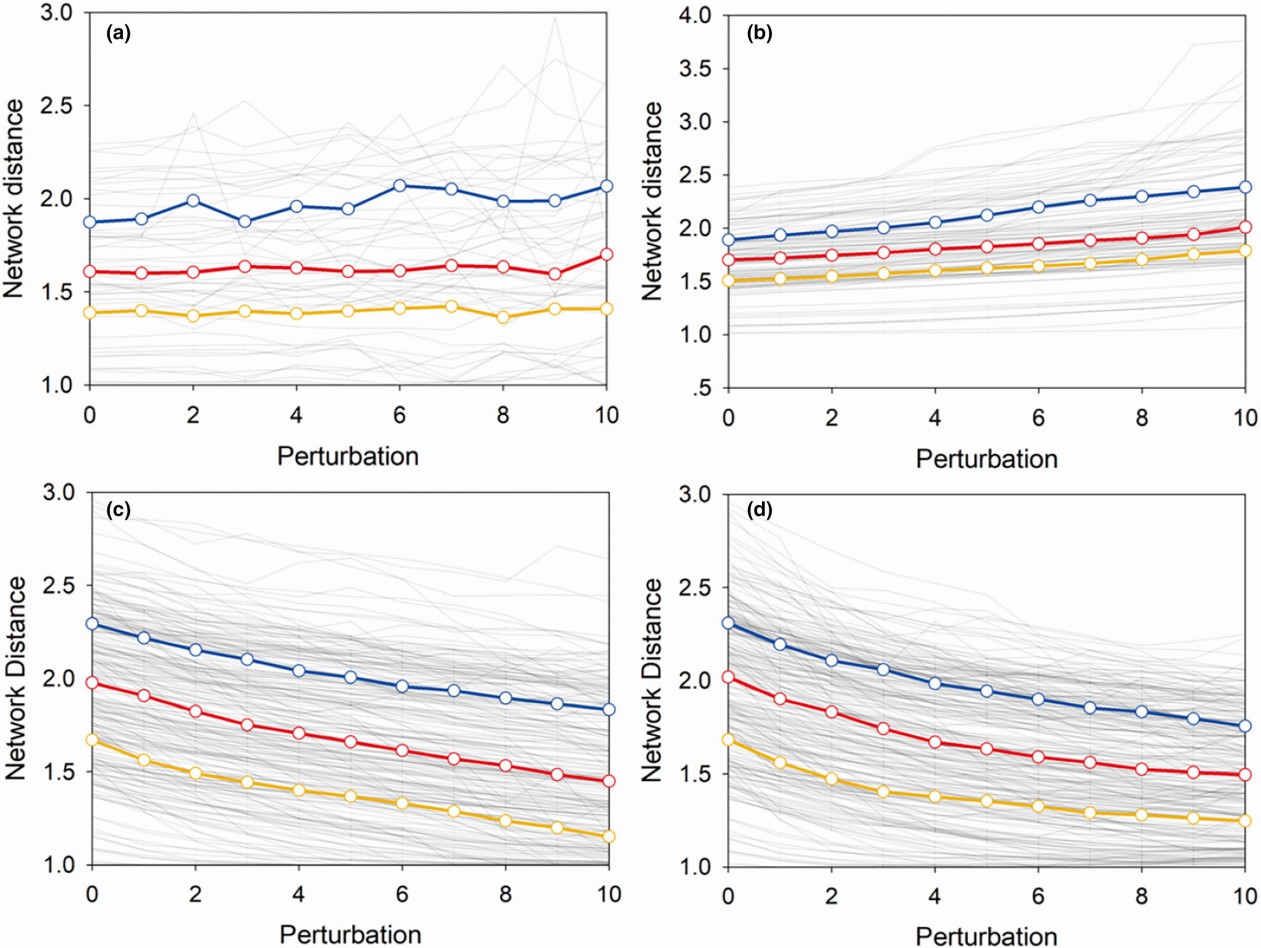
Network simulation. (a) deletion of random species; (b) deletion of large‐degree species; (c) deletion of small‐degree species; (d) deletion of small‐degree species and replacement by large‐degree species. The perturbation in (a–c) reflects the number of species lost, and the perturbation in d represents the number of small‐degree species lost and the number of large‐degree species that replaced them. The grey lines represented each experimental diatom community. The red lines represented the median value under each perturbation, the blue line was the 75th percentile and the yellow line was the 25th percentile.

The simulated random species loss induced no detectable change in the network distance of the empirical diatom networks (Figure 5a). The loss of large‐degree species led to an increase in the network distance (Figure 5b). In contrast, both the loss of small‐degree species and the replacement of small‐degree species by large‐degree species induced a consistent decline in network distance across the full range of perturbations (Figure 5c,d).

## 4 DISCUSSION AND CONCLUSION

In the Chinese diatom dataset, we discovered that altitude and human activity impacted the diatom assemblages of lakes. We found that diatoms showed a high level of richness but a low level of dissimilarity in shallow lakes in eastern China, where the lakes were at low altitudes and had higher human impacts. We found a declining trend in network distance along the gradient of human activities across China, and the richness of diatoms showed a nonlinear relationship with population density (Figure 4). From the simulation, we found that whether the loss of biodiversity caused the decline of network distance was determined by the type of species lost, and that random species loss will not cause structural change, while loss of small‐degree species, or the replacement of small‐degree species by large‐degree species, will cause a reduction in the network distance (Figure 5). These results may suggest a complex relationship between biodiversity change and ecological structure.

In terms of the distribution of diatoms richness in Chinese lakes, we did find that the lakes in low‐altitude regions showed more richness than high‐altitude lakes (Figure 3a), which is consistent with the prediction that a higher‐altitude environment will have a lower biodiversity (Peters et al., 2016). However, the relationship between diatom richness and altitude is not straightforward (Figure 4f). In contrast, diatom richness increased at the lower levels of human impacts intensity, but decreased at higher levels of human activity intensity (Figure 4d). This finding was different from other studies that have concluded that the human impact leads to a decrease in biodiversity (Albert et al., 2021; IUCN, 2019), in which these biodiversity losses mainly refer to aquatic macro‐organisms such as mammals, birds, reptiles and fishes. Here, at least in diatoms in Chinese lakes, the richness will not decrease until it is severely impacted by humans.

Other lines of evidence suggested that communities absorb the impacts of exogenous forcing through losses of canary and keystone species and their replacement by weedy species and that the overall stability in species richness was maintained until the system breaks down at a critical transition point at which biodiversity is greatly reduced (Doncaster et al., 2016; Wang, Xu, et al., 2019). This ordered species replacement in the communities can not be indicated by changes in richness, but it shaped the network structure. In our study, there was a linear correlation between population density and network distance, which species similarity in the communities might be associated with human impacts. In highly disturbed Eastern China (LYB), we simultaneously observed lower pairwise‐*β*‐diversity and network distance. Thus, we were able to conclude that the lake's eutrophication likely induced increasing in generalist species and decreasing in specialist species, and it might be one reason why the changes in richness and network distance were not synchronised. The network simulation further proved that the process of the replacement by generalist species would cause a decline in network distance (Figure 5). Although this process will not cause biodiversity loss, sufficient attention needs to be paid to the changes in community structure caused by it, especially since this process is common in natural ecosystems (Clavel et al., 2011).

The basic idea of network construction in this study is that each species will live in a specific habitat so that they coexist with other species in these similar habitats (Chesson, 2020), so network distance indicates the heterogeneity of both environment and species rather than species competition or mutualistic. Therefore, as in other studies (Delmas et al., 2019; Yuan et al., 2021), the distance of the ecological network in this study measured how quickly perturbations may spread and it evaluated the stability of ecological communities or their resistance to environmental pressures. These pressures are obviously complex in nature ecosystems from either climate change or many kinds of human activities, and possibly cause biodiversity loss (IPBES, 2019). Although environmental pressures will cause many types of biodiversity change (Pereira et al., 2012), our results indicated that not all biodiversity loss will result in stability decline. We found random species loss will not cause changes in network distance, and decreasing specialist species will cause network distance decline. In one aspect, it demonstrated that how biodiversity loss affects ecological stability depends on the type of lost species. It should be pointed out that this study is not aimed at revealing the relationship between diversity and stability (Hooper et al., 2005; Ives & Carpenter, 2007), but rather at exploring the impact of loss of biodiversity on community stability. In another aspect, this result further indicated that the loss of certain species may have obvious effects on ecosystem functions (Zhong et al., 2022).

In summary, for diatom communities in China, we did not find a clear biodiversity decline along the gradient of human activities but observed a good relationship between network distance and the intensity of human impacts. We concluded that the decline of heterogeneity and stability in diatom communities were associated with human activities. The reason for the declines could be due to specialist species loss or specialist species represented by generalist species. Our results suggested that biodiversity loss did not necessarily lead to a decline in ecological stability, which depended on the type of species that was lost. The study did not present all possible scenarios of biodiversity change in real‐world ecosystems, which might be caused by different types of species variations and determined by the type of external drivers.

## AUTHOR CONTRIBUTIONS

**Rong Wang:** Conceptualization (lead); funding acquisition (lead); methodology (lead); writing – original draft (equal); writing – review and editing (equal). **Wenxiu Zheng:** Data curation (equal); formal analysis (equal); funding acquisition (equal); investigation (equal); methodology (equal); writing – review and editing (equal). **Min Xu:** Formal analysis (equal); writing – original draft (equal). **Hui Yang:** Formal analysis (equal); writing – review and editing (equal).

## CONFLICT OF INTEREST STATEMENT

None.

## Data Availability

The data underpinning the results are archived in a Dryad Digital Repository. https://datadryad.org/stash/share/R39qoFIDH6ioRPmvicb9Pr_TOU8jz8XdrAkevYsdHqs.
